# A Concert-Based Study on Melodic Contour Identification among Varied Hearing Profiles—A Preliminary Report

**DOI:** 10.3390/jcm13113142

**Published:** 2024-05-27

**Authors:** Razvan Paisa, Jesper Andersen, Francesco Ganis, Lone M. Percy-Smith, Stefania Serafin

**Affiliations:** 1 Multisensory Experience Lab, Aalborg University Copenhagen, A.C. Meyers Vænge 15, 2450 Copenhagen, Denmark; frga@create.aau.dk (F.G.); sts@create.aau.dk (S.S.); 2 The Royal Danish Academy for Music, Rosenørns Alle 22, 1970 Frederiksberg, Denmark; jesper.andersen@dkdm.dk; 3 Center for Hearing and Balance, Rigshospitalet, 2100 Copenhagen, Denmark; lone.percy-smith@regionh.dk

**Keywords:** melodic contour identification, cochlear implant music, concert research, music perception, hearing impairment music

## Abstract

**Background**: This study investigated how different hearing profiles influenced melodic contour identification (MCI) in a real-world concert setting with a live band including drums, bass, and a lead instrument. We aimed to determine the impact of various auditory assistive technologies on music perception in an ecologically valid environment. **Methods**: The study involved 43 participants with varying hearing capabilities: normal hearing, bilateral hearing aids, bimodal hearing, single-sided cochlear implants, and bilateral cochlear implants. Participants were exposed to melodies played on a piano or accordion, with and without an electric bass as a masker, accompanied by a basic drum rhythm. Bayesian logistic mixed-effects models were utilized to analyze the data. **Results**: The introduction of an electric bass as a masker did not significantly affect MCI performance for any hearing group when melodies were played on the piano, contrary to its effect on accordion melodies and previous studies. Greater challenges were observed with accordion melodies, especially when accompanied by an electric bass. **Conclusions**: MCI performance among hearing aid users was comparable to other hearing-impaired profiles, challenging the hypothesis that they would outperform cochlear implant users. A cohort of short melodies inspired by Western music styles was developed for future contour identification tasks.

## 1. Introduction

A consistent and fundamental component of humans across times and cultures is music, and its powerful multifaceted roles have always been an important factor in the instinctual, emotional, intellectual, and spiritual life of people [1]. Unfortunately, hearing-related sensory impairments can limit the engagement one has with music—a scenario particularly common with cochlear implant (CI) users [2]. However, there is increasing evidence that hearing impaired individuals, and especially CI users, witness an improvement in quality of life as a result of music listening [3,4]. Such benefits range from social and emotional well-being, to better hearing capabilities both in terms of music and speech-related tasks [3,5,6,7].

Before the widespread availability of recorded audio, engaging with music was primarily a live, social, and fundamentally multisensory experience. In this paper, our primary goal was to understand the music hearing performance of adults with various hearing profiles in an ecological setting—a live concert featuring a popular arrangement of drums, electric bass, and a lead instrument. We utilized the melodic contour identification (MCI) test [8] to examine the hearing performance of adults with diverse hearing profiles, including those with bilateral cochlear implants, single-sided cochlear implants (SSCI), bimodal users (individuals with a CI in one ear and a hearing aid in the other), bilateral hearing aid users, and the normal hearing (NH) population. Additionally, we compared our results with previous studies investigating MCI performance [8,9,10,11], to identify potential differences between our ecological approach and other lab-based methodologies.

In recent years, there has been a trend to increase the ecological validity of empirical work in music cognition research [12,13]. One of the areas where this trend has been seen is in the combination of concerts and scientific activities [14,15]. To our knowledge, the only scientific publication connecting concert research and individuals with hearing impairment is presented in [16]. Here, the authors described the execution and evaluation of a concert specifically designed for individuals with CIs. The concert was evaluated through questionnaires and focus group interviews. In the interviews, spatialization was identified as an important element by both normal hearing individuals and individuals with hearing impairment.

### 1.1. Music and Cochlear Implants

Individuals with hearing impairments exhibit a wide range of hearing profiles, capabilities, and perceptual experiences. Such diversity results from various factors such as age, cognitive processing abilities, residual hearing, usage of hearing aids, and musical training [17]. Notably, this variability is even more pronounced among cochlear implant (CI) users.

Cochlear implants are neuroprosthetic devices that partially restore auditory sensations for individuals with sever to profound hearing loss. A CI consists of an external microphone paired with a processor and a limited number of electrodes (18–24, depending on the manufacturer) that are surgically implanted into the cochlea. The frequency allocation typically spans from about 100 Hz (but frequently higher) to 8 kHz, which is divided among the available electrodes implanted in the cochlea. Each electrode is responsible for stimulating a specific part of the cochlear, corresponding to different frequency ranges. This mapping strategy is crucial because it aims to mimic the natural tonotopic organization of the cochlea, where lower frequencies are detected at the apex and higher frequencies at the base [18].

Hearing aids, on the other hand, do not directly stimulate the cochlea but amplify sound frequencies to aid those with residual hearing. Modern digital hearing aids are fairly successful in their ability to amplify specific frequency bands where the user’s hearing loss is most significant. They typically cover a broader frequency range than CIs, often up to 10 kHz, providing a more naturalistic representation of sound [19].

Over the past three decades, cochlear implants have undergone significant advancements, providing over half a million profoundly deaf individuals with partially restored hearing capabilities [20]. The current generation of cochlear implants (CIs) significantly limit pitch and timbre perception. However, there is a raised interest in preserving the residual hearing in the implanted ear, and it seems that music perception is a strong candidate for witnessing improvements through hybrid stimulation, even though acoustic hearing is mostly observed under 300 Hz [21,22,23,24]. Some more radical ideas have looked at re-designing the stimulating interface completely. One such suggestion is to directly implant an array of electrodes in the auditory nerve, bypassing the cochlea completely [25]. This study showed objective benefits of this approach, but so far only animal tests have been conducted. Another direction was suggested by Pinyon et al. [26] who proposed the delivery of drugs to the auditory nerve, in order to stimulate its growth into the cochlear electrodes. An alternative approach considers discarding electrical stimulation and substituting it with optical stimulation [27]. This would imply genetic manipulation of the spiral ganglion, so that they become photo-responsive, as reported by Dieter et al. [28]. However, challenges include energy-efficient delivery and the safety concerns associated with the very early steps of the technology.

Nevertheless, current CI users often struggle to differentiate between sounds in complex auditory environments, such as when multiple instruments are playing simultaneously or in settings with extended reverberations [29,30]. Moreover, even for signals below the CI pitch saturation limit, the representation of fundamental frequencies (F0) in complex sounds is weak, exhibiting difference limens that are ten-times worse than those in normal hearing. Another limitation is the dynamic range for electrical stimulation in CI users, which is only about one-eighth of that available to individuals with normal hearing [31,32]. This significantly impairs their musical listening capabilities and, consequently, their enjoyment of music. Therefore, music perception is generally considered difficult, as all components of music are affected by the implant, namely the musical discrimination abilities, the access to meaning in music, as well as the subjective appreciation of music [2,33]. As a result, CI users tend to attend concerts less frequently [34], and they often dislike music when it is combined with speech, such as in the jingles of TV/Radio news programs or in commercials.

The limited hearing ability of CI users results in a specific set of musical characteristics that correlate to a better music listening experience [35]. Some of these features relate to the harmonic content of the music, where simpler melodies result in a more enjoyable experience [36], some to the rhythmical elements, where percussive components contribute positively to the musical appreciation, as does a lower tempo [37,38,39], while others relate to the instrument’s relative amplitude, and especially the presence and relative loudness of a vocal lead [36,38,39,40]. Moreover, it seems that cochlear implant recipients lack access to pitch interval data when they engage with acoustic stimuli through their speech processing devices [40]. Luckily, there is no significant difference between processing strategies like the standard continuous interleaved sampling (CIS), high definition CIS (HDCIS), and manufacturer-specific ones like MED-EL’s fine structure processing (FSP) with respect to music perception and appreciation. However, the authors in [41] recommended that CI users should be presented with a choice of processing, as short- and long-term performance seemed to vary across participants. Based on the results presented, one could wrongly infer that individuals with cochlear implants would exhibit a strong preference for rap music, as it aligns with the criteria outlined in the aforementioned studies, with loud drums and vocals. However, a study by Gfeller et al. [40] outlined that rap music, followed by classical and jazz, showed the largest decrease in appreciation post-implant. The same authors also documented that music familiarity pre-implant is the most important feature linked to a positive experience [34]. This indicates that CI users have their own unique music listening experience, and comparing it to normal hearing individuals might result in erroneous conclusions.

Music is a compounded stimuli, as it combines temporal and spectral information, as well as complex emotional aspects. With this in mind, our study focused on some of the objectively measurable elements of music: melody, timbre, and combinations of instruments playing simultaneously. One frequently used method to gauge the music hearing performance of cochlear implant users is the melodic contour identification test (MCI) [8]—a task that requires participants to choose the melodic contour of a presented stimuli out of a given set of possible answers (typically 3 to 9). This method relies heavily on the pitch identification abilities of the listeners, without relying on the rhythmical cues presented in music. It therefore assesses some of the most difficult aspects of CI music listening, especially in cases when a masking instrument is present, testing timbre identification aspects as well. Generally, MCI tests highlight the high variability within the CI population, as well as a correlation between music listening training and better test performance, and underline the previously mentioned elements that contribute to a better music listening experience [8,9,42]. One aspect that is poorly disseminated in the MCI literature is the importance of live music in the identification test, as investigated in our study. Nevertheless, there is little or inconclusive evidence that a better MCI performance can be interpreted as a better music listening experience, but one can understand it as one of the enablers of musical enjoyment. One particular study authored by [3] concluded that perceptual performance has some impact on the quality of life and further on self-reported hearing abilities, so interested researchers should be careful in relying only on MCI performance to predict the benefits of music listening mentioned in the Section 1.

### 1.2. The Study

Experiencing music performed live almost always involves complex social aspects, as well as multisensory stimulation (i.e., auditory, visual, and tactile), and both normal and hearing impaired individuals benefit from this. There is evidence that cochlear implant users are better than NH at integrating the different stimuli to create a coherent, singular experience [43]. This process is called multisensory integration, and it describes how different types of stimuli that are happening at the same time are processed together in the the brain and interpreted as one event [44]. Therefore, aiming for ecological validity, in our current study, we presented multisensory stimuli in the form of melodies performed by a live band consisting of professional musicians playing drums, electric bass, and piano or accordion as a lead instrument, in a dedicated concert hall with good acoustic properties. The participants were exposed to four contours (ascending, descending, arching, and undulating) performed by four combinations of instruments—two types of lead with and without the presence of a masker, as well as drums; Section 2 describes the procedure in detail. The overall objective was to investigate MCI performance in a concert scenario across various hearing profiles and with various permutations of common instruments with different spectro-temporal characteristics.

The study was supported by a 30 min free-admission concert that preceded the data collection and acted as an accommodation period, but also as a means of reinforcing the concert scenario environment. The set list for the concert was decided with respect to the preferences of CI users, as reported in [34]. By using this concert scenario, we could recruit participants with various hearing profiles and collect data simultaneously, ensuring that any performance-related differences were consistent across all test groups. The data were collected using an online polling/voting tool called Mentimeter www.mentimeter.com (accessed on 21 May 2024), which was accessed by the participants through their mobile phones.

Based on existing the literature, we expected that participants with different hearing abilities would perform worse than normal hearing users [8], and that individuals with any form of acoustic hearing preserved (i.e., bimodal and hearing aid users) would identify contours better than those relying only on electrical stimulation (bilateral CI, and single-sided implant with deaf counterparts). Additionally, we expected that all groups would perform better than the results from previous studies [9,10], due to the presence of visual stimulation that could give cues about the contours (i.e., hand moves up or down the piano). Another aspect that we investigated was the impact of musically coherent stimuli competing with the MCI performance; the authors of [9] outlined that the presence of a masker decreased the performance in a lab scenario—when the masker had the same frequency as the target stimuli. We wanted to confirm whether their conclusions applied to live music as well, where the masker (the bass line in our case) was played following the chord progression of the lead instrument, but an octave lower—a common occurrence in Western music. Furthermore, our study exposed the participants to two different target stimuli (piano and accordion), with and without the presence of a masker (bass), with the goal of investigating the difference between a percussive stimuli (the piano) and a reed instrument (the accordion) in terms of MCI performance. We expected that melodies played on the piano would result in better MCI scores than the ones played on the accordion when there was no masker, with potentially a similar performance when the bass accompanied the target lead. The rationale was that the broadband energy distribution of the accordion would result in higher pitch confusion than the simpler (harmonically speaking) piano. The choice of stimuli is discussed in detailed in Section 2.4.

## 2. Methodology

In the present experiment, participants were presented with 48 unique melody excepts performed on drums and a target instrument (piano or accordion) with or without electric bass acting as a masker. The general method was inspired by previous MCI studies [3,8,9], but adjusted in order to answer the hypotheses presented in Section 1.2, and where due to the increased complexity presented by the number of independent variables and some missing data entries, a Bayesian analysis approach was preferred [45]. This might result in a difficult direct comparison with previous research, at least in terms of statistical results, but since other aspects of the study had already been altered, that would have not been easily possible anyway.

### 2.1. Experimental Set-Up

The experiment took place in the Royal Danish Academy of Music’s newest chamber music concert hall in Copenhagen, known as “*New Hall*”, which can accommodate up to 90 seated individuals. This venue is equipped with sound-absorbent curtains positioned behind the stage and along the opposite wall, ensuring a favorable acoustic environment with a low reverberation time of approx. 1.2 s—a feature known to be preferred by CI users [30].

The stage was positioned at the same level as the audience, providing a more intimate experience as the band was only a few meters away from some of the participants. The Steinway piano was placed perpendicular to the stage to allow the audience to see the performer’s hands, a crucial visual element, especially when the piano player also performed accordion melodies.

The drum kit included a kick, snare, high-hat, and a ride cymbal, although this cymbal was not used during the experiment and was reserved for the concert. The electric bass was amplified using an *Ampeg Micro-CL Stack* www.Ampeg.com (accessed on 21 May 2024). The overall volume of the band was maintained at a low level, in line with recommendations from the authors of a relevant paper [34].

Before entering the concert hall, each participant had to randomly select an animal name from a bowl containing the 90 most popular animals. They used this pseudonym to ensure anonymity during both the experiment and the data analysis.

### 2.2. Procedure

The data gathering took place at the end of the event, after the attendees had listened to six cover songs performed by the band with a female vocalist. The musical performance lasted about 25 min. For the experiment, participants were asked to identify the melodic contour performed by the band. The task was introduced by the host, who explained what contours are, displayed visual representations of each possible one, and played auditory examples on the piano.

As part of the experiment’s introduction, participants were guided on how to access the data collection platform from their phones. Study assistants were available to provide personal assistance in case of any technical difficulties. Additionally, we provided tablets and phones to audience members who either did not bring their own phones or encountered problems with them.

After all participants had confirmed that their chosen animal name was visible on the projector screen, the experiment commenced. Each contour had a 45 s window for participants to respond, as indicated by a timer on the screen. However, once all registered participants had voted, the experiment conductor manually proceeded to the next question. The audience did not receive any feedback on their performance, but they could see their chosen animal name on the screen after casting their vote. This process was repeated 48 times, with three repetitions for each of the four contours using the following instrument combinations:

- Drums + Piano
- Drums + Bass + Piano
- Drums + Accordion
- Drums + Bass + Accordion

The entire experiment took 22 min.

### 2.3. Participants

**Figure 1:**
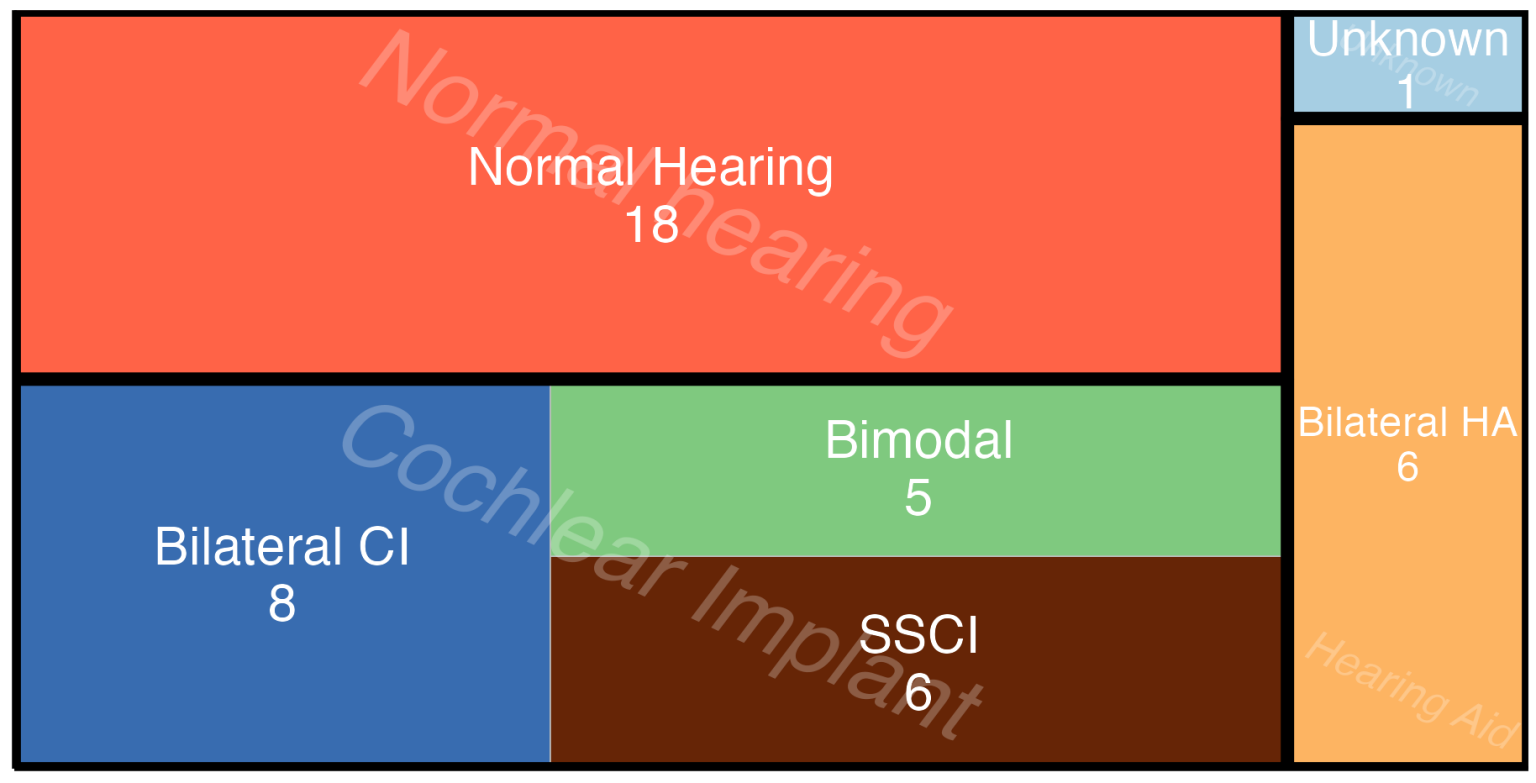
Distribution of hearing profiles for eligible participants; N = 43 (18 NH, 6 Bilateral HA, and 19 CI users), NH = normal hearing, bilateral CI = both ears are implanted, bimodal = CI + hearing aid, SSCI = single-side CI, Bilateral HA = hearing aids in both ears.

A total of 52 individuals participated in the concert, but only 43 responded to more than 80% of the questions and were considered eligible for inclusion in the data analysis. Some participants either did not answer more than one or two questions or left before the experiment began; one participant did not declare their hearing profile; therefore, they were also excluded from the analysis. A distribution of the hearing profiles can be found in Figure 1. In order to increase the ecological validity, no configuration of the hearing assistive devices was requested (i.e., set it to *music mode*, if available). Similarly, it was assumed that the CIs were clinically set to a similarly comfortable loudness level.

Participation was voluntary, and participants were not compensated. The recruitment process utilized various channels, including

1. Advertised on Danish CI social media groups;
2. Local and regional hearing associations (*høreforening*);
3. Local and regional centers for *Education and Communication* (Center for Specialundervisning for Voksne, Roskilde og København);
4. The *Copenhagen Center for Hearing and Balance* at the local hospital (Rigshospitalet);
5. Personal invitations.

A formal event page was created on a ticket management platform www.Eventbrite.dk (accessed on 21 May 2024), where interested participants could reserve their participation free of charge. The event address was advertised on the event poster and flyers. We employed various recruitment channels, with the goal of achieving a true random sampling methodology, while also ensuring a potentially large participation. However, since CI users generally have limited interest in music events, there is a risk of over-representation of individuals actively seeking musical experiences, which may not be representative of the entire CI population.

**Figure 2:**
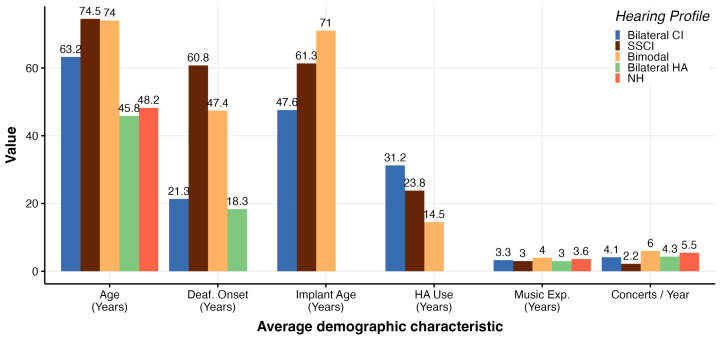
Average demographic information self-reported by participants: “HA use” refers to hearing aid usage before obtaining a cochlear implant, “Music Experience” refers to formal music education or practice prior to implantation (if relevant); NH = normal hearing, Bilateral CI = both ears are implanted, Bimodal = CI + hearing aid, SSCI = single-side CI, Bilateral HA = hearing aid in both ears.

All participants at the concerts were required to complete a survey that covered their hearing abilities, habits, musical experiences, and limited demographic information. Consistent with previous studies, the average age was relatively high (mean = 57.3, sd = 20.8). However, the means for different hearing profiles varied, as illustrated in Figure 2.

We also collected information about the onset of deafness and the age of implantation. Unfortunately, due to some participants’ misunderstandings, this data had to be excluded—some CI users did not identify themselves as deaf, leading them to skip or enter “0” when asked about the onset of deafness. Additionally, some participants confused CI with traditional hearing aids, resulting in inaccurate responses regarding the age of implantation.

### 2.4. Stimuli

Before describing the melodies used in the experiment, it is worth noting that there is no consensus on what constitutes a “melodic contour”. Various perspectives exist when attempting to define this concept, as outlined in [46]. One ethnomusicological perspective that we adopted is to view contours as “*narrating a story*”. This describes the contours as having a musical *start*, an *evolution*, and an *end*, resulting in the four contours utilized in the experiment:

- Ascending: a consistently rising trajectory over a minimum of 12 semitones from the starting note (middle C), with the highest point reached near the end.
- Descending: a continuous downward trajectory over a minimum of 12 semitones from the starting note, with its lowest point near the end.
- Undulating: a mostly flat line oscillating around a single pitch (middle C), fluctuating by a maximum of 4 semitones up or down.
- Arching: rises in pitch for at least 12 semitones from the starting note and then decreases, returning close to middle C. The authors of [46] argued that this type follows the “*logical trajectory of thought*”.

The minimum intervals for each contour were determined based on two studies related to pitch ranking [47,48]. Our aim was to make sure that these intervals would be distinguishable for both hearing aid users and, most importantly, for CI users, who are known to have challenges with pitch identification.

**Figure 3:**
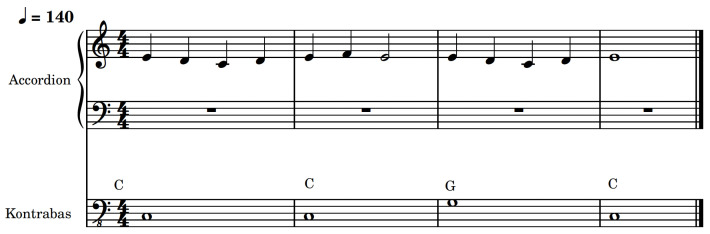
Melody nr. 47 with undulating contour played on accordion and bass; the playing tempo was 70 BPM, but to increase resolution the scores have been written at 140 BPM.

A total of 48 melodies where created to assess the MCI performance of the participants. These monophonic melodies were composed in the key of C major, with an interval structure typical to Western music. The maximum range of the melodies were two octaves, with *middle C* (256 Hz) as the lowest note. The bass line added to some of the melodies was playing simple fundamental notes and, in some cases, a short standard cadenza (I–IV–V–I or I–V–V), and it always followed the same contour as the lead; an example of an undulating contoured one with masker melody present can be seen in Figure 3. To reduce the risk of confounding variables, the melodies included in the experiment were intentionally unfamiliar as previous research identified a correlation between familiarity and pitch ranking [47]. Each melody lasted for 2 bars and was played in a random order at a tempo of 70 BPM, resulting in a stimulus duration of approximately 7.5 s. The melodies can be found in MIDI and audio file at the project’s repository https://github.com/razvysme/MCI-In-Concert (accessed on 21 May 2024). A simple straight drumbeat accompanied each melody, featuring alternating kick and snare hits, along with eighth notes played on the closed hi-hat. The drum groove was present to enhance the “*concert scenario*”, while providing an easily perceivable sense of time, particularly beneficial for CI users.

**Figure 4:**
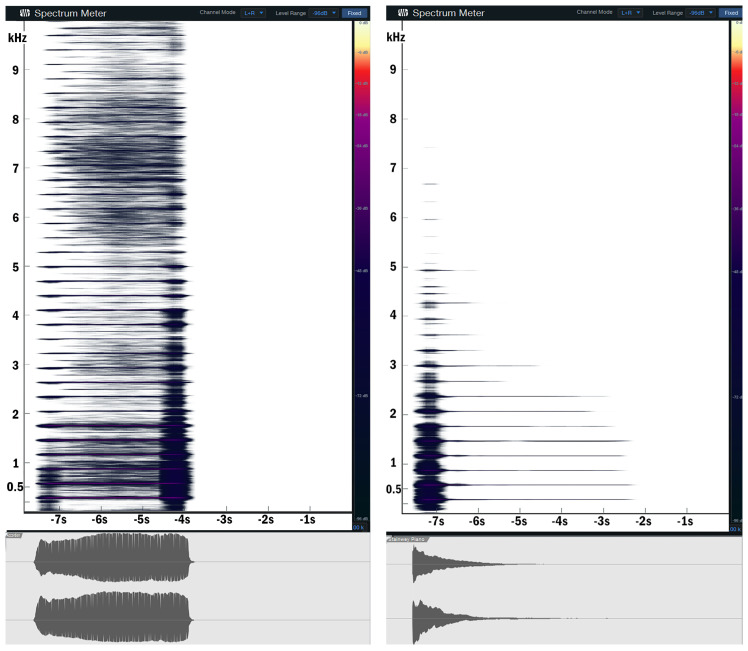
Spectrogram (10—20 kHz) (**top**) and waveform (**bottom**) of an accordion (**left**) and a grand piano (**right**) and playing a single D4 note.

As mentioned before, the melodies were performed on four combinations of instruments: with the lead melody played by two different categories of instruments: piano (Steinway & Sons Grand Piano C) and accordion (Walther Special)—this approach was adopted in order to observe the impact spectra-temporal difference had on the MCI performance. Figure 4 shows the time and frequency domain representation of a D4 note played on a piano and an accordion. The differences in spectral content are very noticeable.

### 2.5. Statistical Analysis

For data analysis, we employed a Bayesian logistic mixed-effects modeling approach [45]. Since the data we collected were categorical, we encoded the discrete factor levels into numeric predictor values, resulting in a contrast/reference coding. The reference levels, representing the *best-case scenario,* were selected based on the existing literature and our judgement. These reference levels included a Normal hearing profile, the instrument combination of Drums + Piano, and the Ascending contour (randomly chosen due to a lack of consensus on which would be easiest to identify). It is important to note that contrast coding means that the results were compared to the reference levels and should be interpreted as deviations from these characteristics.

The dataset inevitably contained missing elements, likely due to participants’ uncertainty in selecting contours or momentary technical difficulties. To address this, we imputed the missing values five times before fitting the model.

The first step was to verify if the demographic information shown in Figure 2 (age, music experience, implantation age, deafness onset age, etc.) would affect the MCI responses (correct or not). To do so, several models were created with and including each variable as fixed effects, followed by a cross-validation using the leave-one-out (LOO) approach [49]. These models were designed to fit binary responses (correct/incorrect) and used a Bernoulli distribution. In the context of binary response modeling, the use of the Bernoulli distribution is synonymous with logistic regression. It allows assessing the probability of a binary outcome based on a set of predictor variables, making it a valuable tool for understanding and predicting categorical responses. No particular prior was used, as there is no model that describes MCI performances; instead the standard priors from the *brms* R library were used.

Once it was established that age and musical experience did not improve the model fitness at all, three models of increasing complexity were created that would account for different fixed effects and interactions between them. The models were as follows:

(a) Considers individual factors (Hearing Profile + Instruments + Contours) separately, and utilizes a random intercept to account for individual variation.
(b) Similar to model A but includes an interaction term (Hearing Profile: Instruments + Contours) to examine the combined effect of Hearing Profile and Instruments. Similarly, it incorporates the same random intercept to address individual variation.
(c) Adopts a more complex approach exploring the potential interaction between all variables simultaneously (Hearing Profile × Instruments × Contours), and as with the other two, accounts for individual variations with random intercepts.

Model A was chosen based on the results of LOO cross-validation, where the expected log pointwise predictive density (ELPD) differences were 0 for model A, −11.1 for B, and −40.9 for model C. A LOO score of 0 indicates that model A provided predictions that were, on average, just as accurate as a model that perfectly fits the data. The negative LOO scores of −11 and −40 for the other models suggested that they performed notably worse in comparison.

After fitting model A, we analyzed the distribution for each level, as well as differences between levels (with respect to the reference levels expected under a contrast coding approach).

Following the completion and discussion of the aforementioned process, we extended the data analysis in an exploratory manner. This extension involved finding differences between hearing profile levels—this change aimed to facilitate a comparison of populations using the two types of hearing-assistive devices: CIs and hearing aids, as well as normal hearing compared to individuals with assistive hearing devices.

## 3. Results

**Figure 5:**
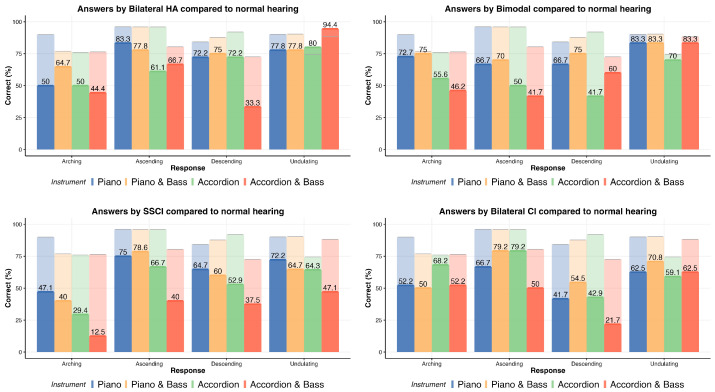
Detailed distribution of correct answers for normal hearing users, separated by contours and instruments played. Normal hearing performance is shown in each plot as the transparent section, while the performance of hearing impaired groups is shown in solid color and numerical value.

The distribution of correct answers for each hearing profile is illustrated in Figure 5. These figures are arranged in the order of anticipated decline in performance, considering that present or residual acoustic hearing is associated with enhanced pitch ranking ability [48]. The data depicted in these figures served as the basis for fitting the models outlined in Section 2.

As anticipated, reduced access to acoustic hearing correlated with lower overall MCI performance. However, noteworthy observations emerged. Participants with normal hearing, for instance, did not achieve perfect scores. In the case of ‘Accordion and Drums’, their correct answer percentage dropped to 60% when the contour was undulating, creating a range of correct answer percentages from 60% to 90%. The average correct answers for the normal hearing population was 79.5%, 65.9% for the bilateral hearing aid users, 49.5% for the bimodal individuals, 47.9% for the single-side CI users, and 55.4% for the bilateral implanted. Additionally, noteworthy observations indicated that among groups of CI users, certain distributions exhibited a trend toward randomness, particularly when the lead melody was played on the accordion. For instance, this was evident in the responses of single-side CI users under the *Drums, Accordion, and Bass* condition, as illustrated in Figure 5. A similar pattern was observed for bilateral implanted individuals under the same conditions, as depicted in Figure 5.

### 3.1. The Effect of Hearing Profiles on MCI Performance

**Figure 6:**
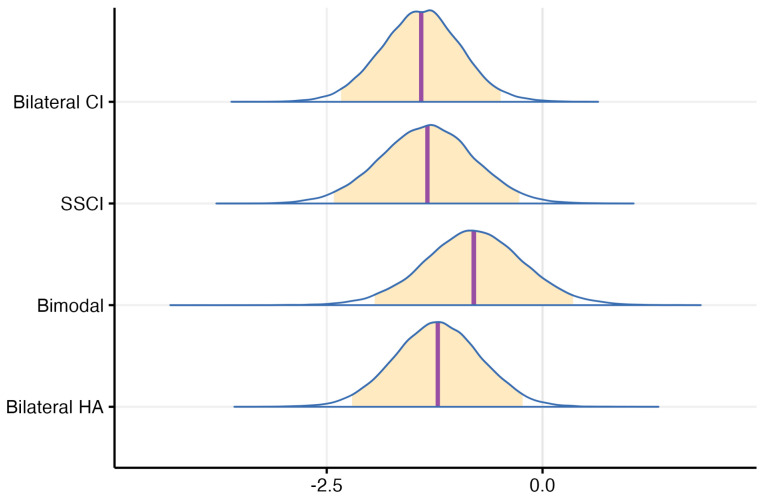
Probability distribution that describes the performance difference from the reference level (0 = Normal hearing) for each hearing profile—orange areas indicate a credibility interval of 95%, purple line indicates the median.

The mixed model detailed in Section 2 was applied to the aforementioned data. Upon closer inspection of the posterior distribution of probabilities (under contrast conditions) for all hearing profiles, a notable trend emerged. The probability of populations utilizing various hearing assistive devices performing worse than the normal hearing group was consistently high—exceeding 95% for all groups and surpassing 95% for the bilateral CI, single-side CI, or bilateral hearing aid groups. Additionally, a visual examination reveals that the differences between the probabilities of groups using hearing-assistive devices were not distinctly pronounced. Instead, they exhibited a comparable median and spread, indicating similar levels of variability, as can be seen in Figure 6.

This aspect was reinforced during a post hoc analysis that compared the pairwise probabilities of hearing profiles. Notably, the probability of the population using a bilateral hearing aid performing worse than those with a bimodal hearing configuration was approximately 73.3%, suggesting a marginal likelihood of inferior performance for the bilateral HA group. Similarly, when comparing bilateral HA with a single-side CI, the probability of the former performing worse was approximately 42.3%. Moreover, contrasting bilateral HA with the bilateral cochlear implant group indicated a probability of around 36.4% for inferior bilateral HA performance.

Furthermore, examining the comparison between bimodal and SSCI profiles, the probability of the bimodal group performing worse was approximately 19.2%. Similarly, when contrasting bimodal with the bilateral CI group, the probability of inferior performance for the bimodal group was around 15.4%.

**Figure 7:**
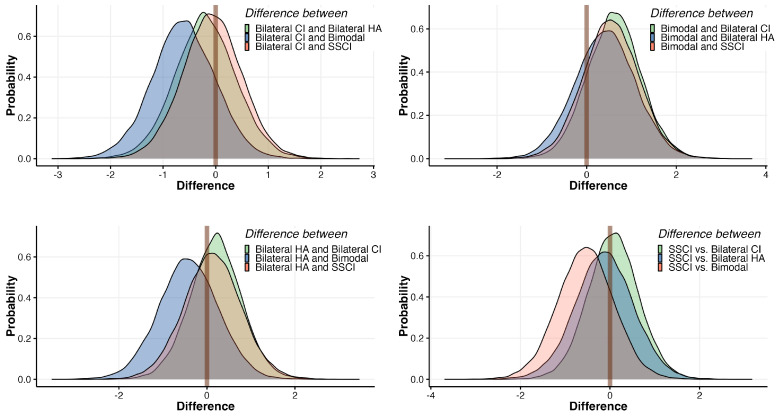
Pairwise probabilities of profile performance differences, with respect to the normal hearing reference level. The mark indicates an “0” equal chance of profile A performing better than profile B.

Lastly, the analysis revealed that, when comparing the SSCI and bilateral CI groups, the probability of the SSCI group performing worse was approximately 44.9%. These percentages underscore the relationships between hearing profiles and their respective probabilities of performance differences (again, with respect to the reference—normal hearing participants). As most of those probabilities were rather close to 50%, with some as low as 15%, we can understand that none of the assistive hearing device using groups performed particularly different than the others. This nuanced insight is summarized in Figure 7, illustrating the pairwise probability differences between all hearing profiles.

### 3.2. The Effect of Instrument Combinations on MCI Performance

**Figure 8:**
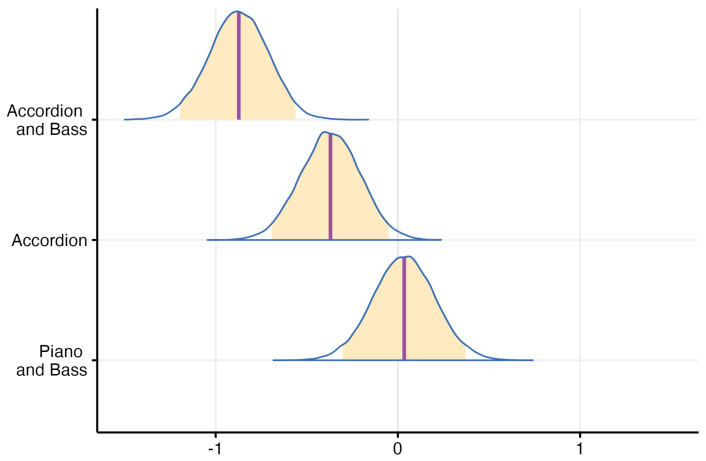
Probability distribution that describes the difference with the reference level (Piano) for each instrument combination—orange areas indicate a credibility interval of 95%, purple line indicates the median.

Looking at the impact of instrument combinations on the MCI performance as shown in Figure 8, the difference between levels was much larger than in the case of hearing profiles. The difference distribution of probabilities were mostly as expected with respect to the *Drums and Piano* reference, with the exception of the *Drums, Piano, and Bass* case that showed a similar change in MCI performance as the one without a masker. Besides that, the probability of a melodic contour played on drums and accordion being correctly identified was lower than the reference with a credibility of over 99%; when adding the electric bass as a masker, the probability moved into practical certainty, according to the model described in Section 2, and fitted the data presented above.

In the post hoc analysis comparing the pairwise probabilities of instrument combinations and their effect on MCI performance, one can clearly observe the same trend. Specifically, when comparing the probability of melodies played on the combination of *Drums, Piano, and Bass* performing worse than those played on the combination of *Drums, Accordion, and Bass*, the calculated probability was precisely 0%, indicating that the melodies played on *Drums, Piano + masker* were easier to identify than similar ones played on the *Drums, Accordion + masker*. Furthermore, when assessing melodies played on the *Drums, Piano, and Bass* combination against those played on the *Drums and Accordion* alone, a similar result of 0.8% was observed, suggesting that the piano as lead instrument was easier to identify, regardless of the presence of a masker. Similarly, when evaluating the probability of melodies played on the *Drums and Accordion* performing worse than those played on the combination of *Drums, Accordion, and Bass*, an even smaller probability of approximately 0.05% was noted, indicating that the presence of bass as a masker negatively impacted the MCI performance.

**Figure 9:**
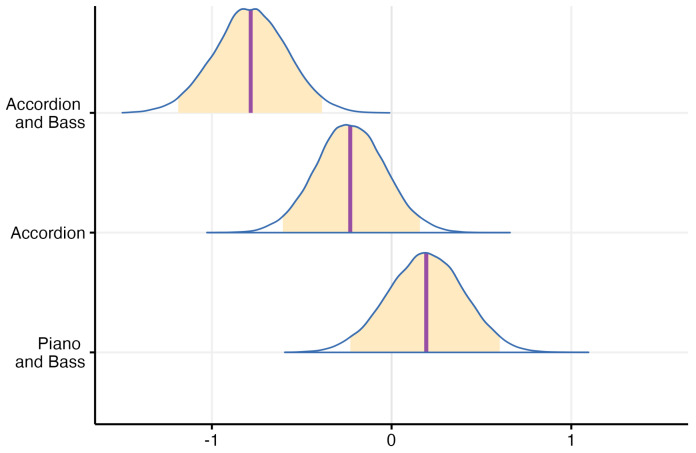
Probability distribution that describes the difference from the reference level (bilateral HA) for each instrument combination, fitted with data excluding the normal hearing population—orange areas indicate a credibility interval of 95%, purple line indicates the median.

Extending the post hoc investigation, we sought to examine whether a similar trend occurred independently for the hearing impaired and normal hearing groups. The regression model, as detailed in Section 2, was re-fitted to the dataset, this time excluding normal hearing individuals, with the *Bilateral Hearing Aid* group as the reference level. The resulting distribution of probabilities is illustrated in Figure 9. Generally, it exhibited a similar trend, indicating a decrease in MCI performance for melodies played on *Drums and Accordion*, and an even more pronounced decline for those played on *Drums, Accordion, and Bass*. However, there was a subtle indication that, for the cumulative hearing-impaired users group, MCI performance may have increased with the presence of the bass. However, the credibility interval hovered around 50%, suggesting insufficient evidence to make definitive statements. Nevertheless, an intriguing aspect of these findings is that the bass as a masker did not negatively influence MCI performance when the lead melody was played on the piano, both for normal hearing and hearing-impaired individuals.

It is worth noting that a considerable portion of our analyses took an exploratory approach. Consequently, the corrections applied for pairwise comparisons tended to be less stringent in these instances, and it is essential to acknowledge that some of the observed findings might not withstand more rigorous correction methods.

## 4. Discussion

The aim of our study was to assess and understand MCI performance across various hearing profiles within an ecologically valid context, without altering common musical practices (e.g., playing in a higher register). We presented melodies designed to resemble popular music fragments commonly encountered in the Western world. Consequently, our results can only be partially compared to studies like [9,10,11]. However, when possible to compare, it appears that we obtained outcomes similar to those reported in the literature. For instance, in [9], the pre-training MCI scores for CI users with piano target stimuli hovered around the 60% correct mark, a similar outcome to our test, as shown in Figure 5. Furthermore, in [10], the normal hearing population did not achieve perfect scores either, mirroring our observations with scores around 90% correct. The scores presented in the previously mentioned studies did not align with ours when masker was presented, but it is worth noting that in our melodies the masker was rarely played at the same time as the target stimuli, and it was at least one octave lower. Nevertheless, the only insight we have on the impact of the masker frequency on MCI performance indicates that it should have no effect (see [11]). However, the authors of the study only tested for maskers with increasingly higher frequencies than the target, making a direct comparison difficult or inaccurate.

### 4.1. Masker Impact on MCI

An interesting observation can be made by comparing our cases with a bass present as masker—the MCI performance seemed unaffected when the target melody was played on the piano, but decreased for the accordion cases. Previous literature indicated that a masker should always have a detrimental impact on MCI performance, a pattern that we did not observe, especially when comparing the results shown in Figure 8 and Figure 9. Such figures indicate an increasing trend in number of correct answers for the *Drums, Piano, and Bass* case compared to *Drums and Piano*. Several factors could have contributed to these results. Firstly, participants may have relied on multisensory cues to identify the contour, such as observing the hands of the piano player. Since the range of motion for the accordion is smaller than the piano, it is possible that less participants could use this visual information to their advantage (due to obstruction from other attendees, poor vision, or not paying attention), while the piano would be visually easier for the majority to understand. Nevertheless, our baseline results were not better than previous MCI studies presenting piano melodies; therefore, we believe that the observed effect was not only due to the multisensory integration.

Secondly, we can analyze the spectral content of the stimulation, as shown in Figure 4, which illustrates the harmonic differences between the instruments. The piano exhibits a sharp, broadband component in the attack that quickly converges on the first 9 harmonics, while the accordion shows a much broader spectral spread, distributed across the analyzed range (10–20 kHz). Additionally, we observe that the inharmonic content is more prevalent in the accordion recording, especially in the 6–8 kHz range, compared to the mostly harmonic nature of the piano. Considering that the electric bass would primarily extend the spectral spread down by an octave for both lead instruments, we can make an informed assumption that there may be a negative correlation between the spectral spread and/or noisiness above the target melody’s frequency and MCI performance. In simpler terms, the higher range of the spectral content could be perceived as disturbing, at least for MCI tests. This interpretation would not contradict the findings of [11], who measured the impact of a masker frequency equal to or greater than the target melody frequency on MCI performance.

Furthermore, it is important to consider that CI users probably relied more on the first or second harmonic for pitch detection, as middle C is 256 Hz and it is not common for implants to stimulate the area of the cochlea responsible for such low frequencies. Hearing aid users usually have the opposite effect, as the perceived frequencies are less affected towards the lower end of the spectrum. With this in mind, the frequency of the electric bass that was playing an octave lower (128 Hz, with overtones similar to that of the piano) should have been perceived even less by CI users, while bilateral HA users should have heard it largely unaffected, but the MCI scores shown in Figure 5 and Figure 9 show an effect of the electric bass on the accordion melodies. This indicates that CI users did perceive the electric bass, but it interacted more with the accordion and it has a broader spectrum. This could be another argument for the fact that noisiness negatively affected CI users’ MCI performance, given that the electric bass would only add energy to an already saturated system.

Another characteristic worth discussing in understanding why the presence of the bass masker did not influence piano MCI performance is the temporal amplitude envelope of the two stimuli. Figure 4 illustrate that the piano has a sharp attack that decays quickly, in contrast to the short and smooth attack of the accordion that sustains for the entire duration of the sound. An electric bass would likely combine the characteristics of both, with a short, broadband attack like the piano and a more pronounced sustain, primarily in the first and second harmonics. In our study, we assumed that the shorter decay of the piano facilitated isolating the melody using temporal cues alone—a hypothesis presented by the authors in [10]. Furthermore, our study indicated that similar envelopes between the masker and the target melody resulted in a lower MCI score, while different envelopes facilitated melody identification, consistent with the findings in [10].

One study that could shed some light on the impact of temporal and spectral characteristics on the MCI scores is [11], where the authors tested six instruments on CI users and ranked them by difficulty from easiest to most difficult as following: organ, glockenspiel, trumpet, clarinet, violin, and piano. This indicates that the temporal characteristics do not impact MCI scores as much as the spectral ones, highlighted by the higher scores for harmonically simpler sounds (organ, glockenspiel) contrasting to richer ones (violin, piano). Unfortunately, this study observed instruments in isolation and not together with a masking instrument, but our findings confirmed this relationship between harmonic complexity and MCI scores, extending the difficulty ranking of instruments, with the accordion being most challenging for melodic contour identification.

To summarize, contrasting MCI performances between piano and accordion when a masker was present suggests a combination of factors. Participants likely relied on visual cues more for the piano, and spectral analysis indicated differences in harmonic characteristics that may have influenced performance. The accordion’s broader spectral spread, particularly in the 6–8 kHz range, could have negatively impacted the MCI. Furthermore, differences in temporal envelopes between the piano and accordion, compounded by potential effects from the electric bass, contributed to the complex relationship. Nevertheless, this intricate spectro-temporal relationship between the masker and target melody requires further investigation to confidently confirm its impact on MCI performance.

### 4.2. Access to Acoustic Hearing and MCI

One of the hypotheses stated in the Introduction was that access to acoustic hearing correlates positively with MCI performance—a statement that proved difficult to confirm in our study, because bilateral HA participants performed equally well as CI users. Pitch ranking is generally accepted to be most difficult for CI users, and a previous study indicated that acoustic hearing facilitates melody and timbre recognition [50], indicating that hearing aid users should perform better than CI users in MCI tests. Nevertheless, we observed a decrease in performance that correlated negatively with access to acoustical hearing (Bilateral CI and SSCI individuals performed marginally worse than bimodal and bilateral HA individuals), but there was no statistical difference between groups. Given the low population number in each group, this conclusion would not withstand rigorous analysis relying exclusively on our data. Unfortunately, there are no known MCI studies that investigated hearing aid users for us to compare our results with, as most of the research focused on CI subjects.

One potential reason that could explain our observations regarding bilateral HA subjects is that the challenges of attending a concert might have outweighed the potential benefits associated with access to acoustic hearing. This could be attributed to the novel acoustic environment, the presence of other individuals, the noises that comes with being in a public space, or the increased cognitive load resulting from processing all these factors combined. What we assume is that the complexity of a concert scenario was detrimental to the hearing aid users, and the eventual pitch ranking benefits associated with acoustic hearing were not relevant.

### 4.3. Further Discussion

The average age of the populations in each of the hearing profile groups was not consistent, with CI users being significantly older than the bilateral HA and normal hearing users; the difference was up to 20 years. Based on our data, age did not impact the model significantly, as confirmed by comparing models fitted including “Age” as a predictor to models that did not.

A similar comparison was made between models that accounted for the question number, based on the fact that some participants expressed being tired at the end of the study. While the experiment was long, there was no benefit when including question order in the model, suggesting that the participants’ remarks did not have an impact on their performance. Nevertheless, we suggest a shorter test is used in similar scenarios, to avoid the risk of fatigue.

Lastly, our melodies were accompanied by drums for all conditions, leading to the question of whether drums can be considered a masker. While technically, the drums serve as competing stimuli, we believe that their low volume and the simplicity of the groove did not significantly impact the MCI performance. This assumption is supported by similar results being obtained in our experiment for the “Piano and Drums” condition, showing comparable outcomes for both CI and NH populations as those reported in studies by [9,10,11].

### 4.4. Limitations

Our findings, specifically those related to the comparison between results from the *Drums, Piano, and Bass* and *Piano and Bass*, may be strongly dependent on the specific setup used in the study, primarily the piano position and the relative amplitude of the instruments. Since we aimed to preserve the concert experience as realistically as possible, we did not control the participants’ placement in the room. On one hand, it is possible that some participants had a better acoustic experience by sitting in the middle of the room, while on the other hand some participants might have had better visual cues by being located closer to the musicians. Throughout our data analysis, we have assumed that the distribution across seats was random for all groups, and this is encapsulated in the model by using random intercepts for each participant, but we have not validated this assumption.

Another limitation that resulted from our aim to preserve the concert experience was related to the recruitment process, and thus the relative number of participants for each hearing profile. Even though we employed standard methods for addressing missing data and uneven populations, the relatively small sample size for each group reduced the confidence in the results presented.

## 5. Conclusions

In this article we presented a MCI study in an ecologically valid context—a concert, with 43 participants with five hearing profiles: normal hearing, bilateral hearing aids, bimodal(hearing aid and cochlear implant), single-side cochlear implant, and bilaterally implanted. Our results mostly confirmed previous studies that were conducted in laboratories, but we observed some differentiating factors as well. Based on the data collected, we concluded that presenting an accompanying electric bass stimuli to a piano melody did not affect the MCI performance, unlike for an accordion melody. Furthermore, the results indicated that it was more difficult to follow melodies played on the accordion compared to ones on the piano, especially when the melodies were accompanied by a groove played on the electric bass. Lastly, we collected data on the MCI performance of hearing aid users, and contrary to our initial hypothesis, they did not perform better than cochlear implanted subjects.

## Data Availability

The original data presented in the study are openly available in Github at https://github.com/razvysme/MCI-In-Concert, accessed date 21 May 2024.
